# A potentially abundant junctional RNA motif stabilized by m^6^A and Mg^2+^

**DOI:** 10.1038/s41467-018-05243-z

**Published:** 2018-07-17

**Authors:** Bei Liu, Dawn K. Merriman, Seung H. Choi, Maria A. Schumacher, Raphael Plangger, Christoph Kreutz, Stacy M. Horner, Kate D. Meyer, Hashim M. Al-Hashimi

**Affiliations:** 1 Department of Biochemistry, Duke University School of Medicine, Durham, NC 27710 USA; 2 Department of Chemistry, Duke University, Durham, NC 27710 USA; 3 Institute of Organic Chemistry and Center for Molecular Biosciences Innsbruck (CMBI), University of Innsbruck, 6020 Innsbruck, Austria; 4 Department of Molecular Genetics and Microbiology, Duke University School of Medicine, Durham, NC 27710 USA; 5 Department of Medicine, Duke University School of Medicine, Durham, NC 27710 USA

## Abstract

*N*^6^-Methyladenosine (m^6^A) is an abundant post-transcriptional RNA modification that influences multiple aspects of gene expression. In addition to recruiting proteins, m^6^A can modulate RNA function by destabilizing base pairing. Here, we show that when neighbored by a 5ʹ bulge, m^6^A stabilizes m^6^A–U base pairs, and global RNA structure by ~1 kcal mol^−1^. The bulge most likely provides the flexibility needed to allow optimal stacking between the methyl group and 3ʹ neighbor through a conformation that is stabilized by Mg^2+^. A bias toward this motif can help explain the global impact of methylation on RNA structure in transcriptome-wide studies. While m^6^A embedded in duplex RNA is poorly recognized by the YTH domain reader protein and m^6^A antibodies, both readily recognize m^6^A in this newly identified motif. The results uncover potentially abundant and functional m^6^A motifs that can modulate the epitranscriptomic structure landscape with important implications for the interpretation of transcriptome-wide data.

## Abstract (web summary)

*N*^6^-Methyladenosine (m^6^A) is a post-transcriptional RNA modification that modulates RNA structure through a destabilization of m^6^A base pairing. Here the authors use NMR and UV melting experiments and show that m^6^A can also stabilize m^6^A–U base pairs and global RNA structure when positioned adjacent to a 5ʹ bulge.

## Introduction

**Fig. 1:**
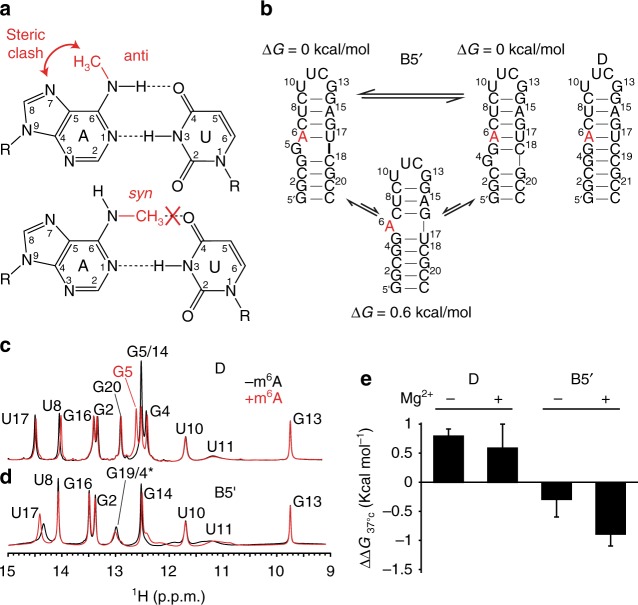
m^6^A stabilizes junctional A–U base pairs with 5ʹ bulge. **a** The methyl group in m^6^A destabilizes A–U pairing through steric contacts. **b** Design of an m^6^A-dependent RNA structural switch. Secondary structures and free energies computed using the MC-Fold web server^39^. The modified adenine (A6) is highlighted in red. **c**, **d** Comparison of ^1^H NMR spectra of unmodified and m^6^A6 substituted (**c**) D and (**d**) B5ʹ recorded in 15 mM sodium phosphate, 25 mM NaCl, 0.1 mM EDTA and 3 mM Mg^2+^ at pH 6.4 and 10 °C. **e** Impact of methylation on the thermal stability of the hairpins with and without 3 mM Mg^2+^. Shown are the differences in the free energy of melting between methylated and unmodified RNA, i.e., ΔΔ*G* *=* Δ*G*(methylated) − Δ*G*(unmodified). The uncertainty reflects the standard deviation from at least three measurements (see Supplementary Table 1)

N^6^-methylated adenosine (m^6^A) is the most abundant internal RNA modification in eukaryotic mRNAs^1–3^ and long non-coding RNAs (lncRNA)^4,5^ and is also found in viral RNAs^6–8^. The modification is dynamically regulated by methyl transferase^9–11^ and demethylase enzymes^12,13^ that are linked to human disease^14,15^. It frequently occurs at the 3ʹ and 5ʹ untranslated regions of mRNA where it can influence RNA splicing^16,17^, mRNA nuclear exportation^13,18^, RNA decay^19^, and translation initiation^20,21^. The modification is implicated in a growing number of processes including stem cell fate determination, stress response, DNA damage repair, microRNA biogenesis, lncRNA-mediated transcription repression, viral infection, and in the mechanisms of cancer^22–25^.

m^6^A is thought to exert its biological effects through two general mechanisms. First, it can help recruit proteins that regulate mRNA fate by influencing splicing^16^, export^26^, decay^19,27^, and translation initiation efficiency^20,27^. Many of these m^6^A reader proteins contain YTH domains, which specifically recognize the methyl group^5^. Second, m^6^A can exert biological effects by modulating RNA structure. In particular, studies have shown that m^6^A destabilizes A–U base pairing and RNA duplexes by 0.5–1.7 kcal mol^−1^ ^28,29^. This destabilization has been proposed to occur due to steric contacts between the *N*^6^-methyl group and the base^28^ (Fig. 1a). Studies have shown that m^6^A can promote RNA melting and thereby enhance binding of single-stranded RNA binding proteins^30–32^. The modification can also disrupt non-canonical A–G base pairs (bps) required for protein binding^33^. Presence of m^6^A in mRNA also impedes tRNA accommodation within the ribosome and translation-elongation most likely by destabilizing A–U bps^34^. Interestingly, while transcriptome-wide RNA structure mapping data indicate that m^6^A destabilizes RNA structure in vitro and in vivo^28,35^, the destabilization is not observed at the m^6^A nucleotide per se, but rather, at the immediate 5ʹ neighbor which favors single-stranded conformations in methylated RNA^28^. The mechanism by which m^6^A destabilizes its 5ʹ-neighbor is currently unknown.

Fewer studies have examined the potential for m^6^A to stabilize RNA even though there is precedent for this in the literature. Prior studies have shown that when placed at dangling ends of duplexes^28^ or in apical loops^29^, m^6^A stabilizes RNA by 0.1–1.2 kcal mol^−1^ most likely due to favorable stacking interactions between the methyl group and adjacent bases. Here, by attempting to harness the destabilizing effects of m^6^A in the design of an RNA secondary structural switch, we discovered a bulge motif that is stabilized by m^6^A in a Mg^2+^-dependent manner. This motif is recognized by the YTH domain and m^6^A antibodies and can potentially help explain the global impact of m^6^A on RNA structure observed in transcriptome-wide studies.

## Results

### m^6^A stabilizes a junctional A–U base pair

Our initial motivation was to examine whether the destabilizing effects of m^6^A on A–U base pairing could be harnessed to induce a change in RNA secondary structure. We designed an RNA hairpin (B5ʹ, Fig. 1b) containing the most common^4,5^ (GGm^6^ACU) m^6^A consensus sequence (DRACH, where D denotes A, G, or U; R is A or G; and H is A, C or U)^36–38^. This sequence is predicted^39^ to fold into two iso-energetic secondary structures in which the m^6^A forms an A–U bp either at the junction next to a bulged guanine or deeper within the upper helix (Fig. 1b). The sequence is also predicted to form an alternative conformation in which the m^6^A is bulged out and in which the 5ʹ neighboring guanine forms a non-canonical G–U wobble (Fig. 1b). This alternative conformation is predicted to be destabilized by 0.6 kcal mol^−1^, which is within the range of m^6^A destabilization of A–U bps in duplex RNA^28,29^. Thus, we reasoned that m^6^A could destabilize the junctional A–U bp and promote formation of the alternative conformation containing the G–U mismatch, which can be readily detected by 1D ^1^H NMR^40^. As a control, we also carried out experiments on a corresponding RNA duplex (denoted D), in which a cytosine nucleotide was included to pair with the bulged guanine (Fig. 1b). Experiments were carried out in the presence of 3 mM Mg^2+^.

The m^6^A substitution resulted in small perturbations in NMR spectra of the control duplex D (Fig. 1c). We observed the U17-H3 imino resonance of the m^6^A6 partner (Fig. 1c), which also has a nuclear Overhauser effect (NOE) cross peak with the amino group of m^6^A6 (Supplementary Fig. 1). This indicates that m^6^A6 forms a m^6^A6-U17 Watson-Crick bp stabilized by two H-bonds, as described previously for other RNA duplexes containing m^6^A^28^. Two NOE cross peaks with comparable intensities were observed between the m^6^A6 methyl proton and the base m^6^A6-H8 and m^6^A6-H2 (Supplementary Fig. 1). This indicates that the amino group either deviates from a perfectly *anti* conformation or rapidly (on the NMR chemical shift timescale) exchanges between the *anti* and *syn* conformations (see Supplementary Note).

The NMR spectra show that unmodified B5ʹ folds into a conformation with the predicted junctional A6-U17 Watson-Crick bp, bulged G5, and with G4 either forming a weak junctional bp or a bulge (Fig. 1d and Supplementary Fig. 2a, c). The m^6^A6 substitution did not lead to the predicted transition to the alternative conformation, as we did not observe the upfield shifted (10–12 ppm) imino resonances characteristic of G–U wobbles (Fig. 1d and Supplementary Fig. 2a). On the contrary, the substitution sharpened the U17 imino resonance, consistent with stabilization of the junctional Watson-Crick m^6^A6-U17 bp (Fig. 1d and Supplementary Fig. 2a). This together with uninterrupted NOE distance-based connectivity between U17 and G16 indicate that the methylation stabilizes the helical structure (Supplementary Fig. 3b). As in D, the NMR data indicate that while m^6^A6 forms a m^6^A6-U17 Watson-Crick bp, the methyl group deviates from a perfectly *anti* conformation (Supplementary Fig. 1 and see Supplementary Note). Therefore, counter to our predictions, and to the behavior observed in duplexes, m^6^A locally stabilizes the junctional A–U bp in B5ʹ.

### m^6^A globally stabilizes B5ʹ in a Mg^2+^ dependent manner

While the NMR data indicate that the m^6^A modification locally stabilizes the junctional A–U bp, this provides little information regarding the effect of the modification on overall RNA stability. We therefore used UV melting experiments to examine the impact of the m^6^A6 on RNA stability. Consistent with prior studies^28,29^, m^6^A destabilized D by 0.6 ± 0.4 kcal mol^−1^ at 37 °C. In stark contrast, m^6^A stabilized B5ʹ by 0.9 ± 0.2 kcal mol^−1^ at the same temperature (Fig. 1e).

To further examine the basis for m^6^A-dependent stabilization, we carried out UV melting and NMR experiments in the absence of Mg^2+^. The modification destabilized D by a slightly greater amount (~0.8 kcal mol^−1^) in the absence of Mg^2+^ (Fig. 1e). This was accompanied by broadening of the imino resonances in the absence of Mg^2+^ (Supplementary Fig. 3d), consistent with the destabilization observed using UV. In contrast, in the case of B5ʹ, the degree of m^6^A stabilization decreased significantly from 0.9 ± 0.2 kcal mol^−^^1^ to 0.3 ± 0.3 kcal mol^−1^ in the absence of Mg^2+^ (Fig. 1e). NMR spectra, including data for site-specifically labeled B5ʹ (Supplementary Fig. 2a), indicate that in the absence of Mg^2+^, unmodified B5ʹ folds into many distinct and slowly (on the NMR chemical shift timescale) exchanging conformations. The U17 imino resonance in methylated B5ʹ is significantly broadened, consistent with local melting of the m^6^A6-U17 bp in the absence of Mg^2+^ (Supplementary Fig. 2a and Fig. 3d). Here, the NOE data indicate that the methyl group adopts an *anti* conformation (Supplementary Fig. 1) and that G5 forms a G5-U17 mismatch (Supplementary Fig. 2a). Thus, the addition of Mg^2+^ induces a conformational switch relative to the structure without Mg^2+^ by stabilizing the junctional m^6^A6-U17 bp with a 5ʹ neighboring G5 bulge.

### Structural requirements for m^6^A stabilization

Previous studies have shown that when placed at dangling ends, m^6^A stabilizes RNA duplexes by ~0.1–1.2 kcal mol^−1^^28,29^. This has been attributed to favorable stacking and hydrophobic shielding of the methyl group. Interestingly, this stabilization is greater when m^6^A is placed at the 5ʹ (~ 1.2 kcal mol^−1^) versus 3ʹ (~ 0.1–0.8 kcal mol^−1^)^28,29^ end of the duplex probably due to stronger stacking with the 3ʹ neighbor. Based on the thermodynamic parameters obtained from the UV experiments (Supplementary Table 1), the observed m^6^A-mediated stabilization of B5ʹ is driven by more favorable enthalpy, which is consistent with formation of favorable structural interactions. In particular, the 5ʹ neighboring guanine bulge could provide m^6^A enough “wiggle room” to form m^6^A–U WC bps in which m^6^A optimally stacks with the 3ʹ neighbor. This may require an unusual backbone conformation at the bulge, which is stabilized by Mg^2+^. Such conformations with optimal stacking may not be easily accommodated within the more rigid duplex interior, resulting in a net destabilization of the duplex.

**Fig. 2:**
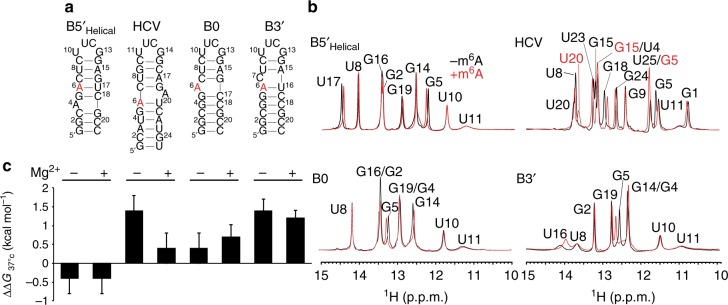
Structural requirements for m^6^A stabilization of junctional A–U base pairs. **a** Hairpin constructs that vary the secondary structural context of m^6^A. Secondary structures are predicted using MC-fold^39^ and supported by NMR data (see Supplementary Fig. 3c). The HCV sequence is derived from the base of stem loop 1 (SL1) in the 3ʹ end of HCV genome (nt 9633-9642 linked by UUCG to nt 9668-9678) from the genotype 2A JFH1 strain (GeneBank accession: AB047639 [https://www.ncbi.nlm.nih.gov/nuccore/AB047639]). **b** Comparison of ^1^H NMR spectra of unmodified and m^6^A6 substituted hairpin constructs recorded in 15 mM sodium phosphate, 25 mM NaCl, 0.1 mM EDTA and 3 mM Mg^2+^ at pH 6.4 and 10 °C. **c** Impact of methylation on the thermal stability of the hairpins with and without 3 mM Mg^2+^. Shown are the differences in the free energy of melting between methylated and unmodified RNA, i.e., ΔΔ*G* *=* Δ*G*(methylated) − Δ*G*(unmodified). The uncertainty reflects the standard deviation from at least three measurements (see Supplementary Table 1)

To test the structural requirements for m^6^A stabilization, we varied the position and structural context of m^6^A (Fig. 2a) using three variants of the B5ʹ RNA (B5ʹ_helical_, B0, and B3ʹ). We also examined a model RNA (HCV) derived from a naturally occurring m^6^A site identified in the 3ʹ UTR of the hepatitis C virus (HCV) genome^8^. In HCV, the m^6^A site is at the junction but on the strand opposite to the bulge. We performed NMR analysis in the presence of at 25 mM NaCl and 3 mM Mg^2+^ and confirmed that for all variants the methylated and unmodified constructs fold into their predicted secondary structures (Fig. 2b and Supplementary Fig. 3c).

Decreasing the conformational freedom available to m^6^A by moving the m^6^A–U bp one base pair deeper into the upper helix (B5ʹ_helical_, Fig. 2a) decreased the m^6^A dependent stabilization from 0.9 ± 0.2 kcal mol^−1^ to 0.4 ± 0.4 kcal mol^−1^ (Fig. 2c). Decreasing the conformational freedom by placing m^6^A opposite rather than immediately adjacent to the bulge in HCV resulted in a net destabilization by 0.4 ± 0.4 kcal mol^−1^(Fig. 2c). NMR spectra show that in both cases, the modification does not locally stabilize the m^6^A–U bp again possibly due to the lack of conformational freedom needed for optimal stacking (Fig. 2b).

Disrupting stacking with the 3ʹ neighbor through placement of a bulge 3ʹ to m^6^A in B3ʹ also resulted in a net destabilization by ~1.2 kcal mol^−1^(Fig. 2c). Interestingly, although m^6^A globally destabilizes B3ʹ, it did locally stabilize the m^6^A6–U16 bp as evidenced by sharpening of the U16 imino resonance (Fig. 2b). Finally, placement of m^6^A in a bulge position destabilized B0 by ~0.7 ± 0.3 kcal mol^−1^(Fig. 2c). Here, the NMR spectra (Supplementary Fig. 1) indicate that the methyl group adopts a *syn* conformation, consistent with prior studies of single-stranded RNA^41^, and that the modification induces the flipping out of the bulge adenine (Supplementary Fig. 4b). Loss of intra-helical stacking with the adenine bulge could explain the m^6^A-induced destabilization of B0. This result shows that the destabilizing effects of m^6^A are not limited to bps in duplexes but can extend to bulges as well.

Similar results were obtained in the absence of Mg^2+^ with the exception of HCV for which the destabilization was more significant in the absence (1.4 ± 0.4 kcal mol^−^^1^) versus presence (0.4 ± 0.4 kcal mol^−1^) of Mg^2+^ (Fig. 2c). Together, these studies indicate that the observed m^6^A dependent stabilization requires a specific motif in which an m^6^A–U bp is neighbored by a 5ʹ guanine bulge.

### Abundance of junctional m^6^A base pairs transcriptome-wide

Prior transcriptome-wide nuclease mapping studies of methylated RNA from human cells revealed a clear transition in the average RNA structure at the DRACH motif upon methylation^28^. In the methylated RNA, the purine nucleotide immediately 5ʹ to m^6^A had a much higher probability of being single-stranded whereas the methylated adenine had a slightly higher probability of being double-stranded^28^. The two nucleotides immediately 3ʹ to m^6^A showed a strong tendency to be paired. Similar results were observed with in vitro and in vivo SHAPE experiments^35^ although the m^6^A site showed slightly higher reactivity, indicating a greater tendency to be unpaired. These results indicate that methylation thermodynamically biases m^6^A in cellular mRNAs to be located between single-stranded unpaired RNA and adjacent to helices^28^.

Remarkably, the m^6^A stabilized B5ʹ motif identified in this work captures the unique conformational signatures induced by m^6^A in transcriptome-wide studies. The motif partially protects m^6^A by positioning it at a junction, exposes the 5ʹ guanine bulge, while the 3ʹ neighbors are helical. The abundance of the 5ʹB-[m^6^A–X] (where B is any bulge nucleotide and X is any nucleotide) motif could increase significantly on methylation because of two combined effects. On the one hand, m^6^A destabilizes bps in duplexes as well as bulges by as much as ~1.7 kcal mol^−1^. On the other hand, it can stabilize the 5ʹB-[m^6^A–U] motif by ~1 kcal mol^−1^. The combined contribution of ~3 kcal mol^−1^ could significantly bias the RNA folding landscape away from structures in which m^6^A is embedded within duplexes and bulges toward 5ʹB-[m^6^A–U] and perhaps other 5ʹB-[m^6^A–X] motifs and to a degree that help account for the observed changes in transcriptome-wide structure-mapping data. Such a strong bias is required to explain the changes in RNA conformation induced by methylation especially since m^6^A occurs sub-stoichiometrically in most RNAs^5,42^.

**Fig. 3:**
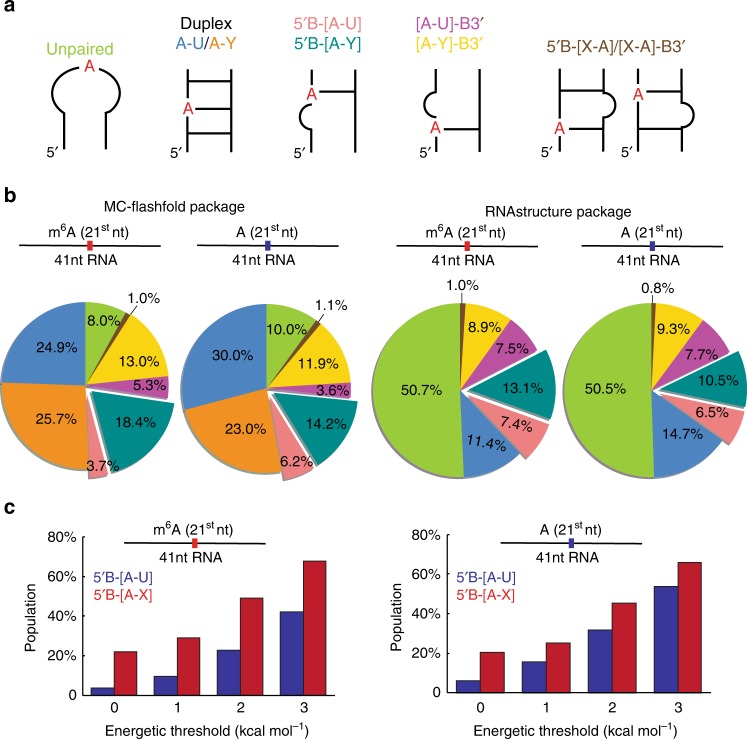
Predicted secondary structures of m^6^A containing RNA sequences. **a** Predicted RNA secondary structures were classified into the following motifs: Junctional Watson-Crick m^6^A–U or mismatch m^6^A–Y (Y denotes A or C or G.) with the bulge located 5ʹ (5ʹB-[A–U] and 5ʹB-[A–Y]) or 3ʹ ([A–U]-B3ʹ and [A–Y]-B3ʹ) to m^6^A; or with the bulge located 5ʹ or 3ʹ (5ʹB-[X-A]/[X-A]-B3ʹ, X denotes A or C or U or G) to m^6^A partner nucleotide. **b** Distribution of the lowest energy predicted structures for 140,574 m^6^A sites identified using individual-nucleotide-resolution cross-linking and immunoprecipitation (miCLIP)^43^ in the human transcriptome (hg19) were predicted using RNAstructure^44^ and MC-Flashfold^39^. Secondary structures were predicted for RNA sequences of 41-nt long with m^6^A (or A) in the middle. Also shown are corresponding predictions for 140,574 unmodified adenine sites selected randomly from the same human transcriptome. **c** Population of m^6^A and A sites predicted (MC-Flashfold) to fold into the 5ʹB-[A–U] and 5ʹB-[A–X] conformation within different energetic thresholds relative to the lowest energy structure

To test this hypothesis, we asked what fraction of the 140,574 m^6^A sites that have been mapped throughout the human transcriptome^43^ are predicted to fold into the 5ʹB-[m^6^A–U] or related 5ʹB-[m^6^A–X] motifs as the energetically most favorable secondary structure. We then asked what additional sites are predicted to fold into the 5ʹB-[m^6^A–U] or 5ʹB-[m^6^A–X] motifs as higher energy conformations that are within the ~3 kcal mol^−1^ energetic threshold. These conformations could be remodeled by m^6^A such to adopt the 5ʹB-[m^6^A–U] or 5ʹB-[m^6^A–X] motifs as the most energetically stable conformation. In particular, we used MC-Flashfold^39^ to predict secondary structures for 41 nt fragments chosen such that the m^6^A residue was positioned in the middle. Predicted RNA structures were classified according to the secondary structural context and position of m^6^A (Fig. 3a). As a control, secondary structures were also predicted for 140,574 randomly selected RNA sequences from the same transcriptome that do not contain the m^6^A consensus sequence.

Approximately 4 and 22% of the m^6^A sites are predicted to fold into the 5ʹB-[m^6^A–U] or 5ʹB-[m^6^A–X] motif respectively as the most energetically favorable secondary structure (Fig. 3b). Similar abundances were obtained for the control unmodified sequences (Fig. 3b), indicating that sequences selected for methylation do not have an enhanced propensity to fold into the 5ʹB-[A–X] motif relative to random sequences. Similar results were also obtained when using a different RNA structure prediction program (RNAstructure^44^) and when varying the position of m^6^A or length of the sequences subjected to structure prediction (Fig. 3b and Supplementary Fig. 5a).

A much larger fraction of m^6^A sequences (~ 42 and ~ 68% for 5ʹB-[m^6^A–U] and 5ʹB-[m^6^A–X], respectively) are predicted to fold into the junctional motif with energies < 3 kcal mol^−1^ relative to the lowest energy structure (Fig. 3c). Similar results were obtained when predicting structures using RNAstructure^44^ (Supplementary Fig. 5b). These higher energy structures could become the most stable structures upon methylation. While a similar increase in abundance is observed for the control unmodified sequences (Fig. 3c), indicating that the potential to form 5ʹB-[A–X] motif is not sequence dependent, these sequences are less likely to be methylated and to experience the energetic bias. Consequently only 20% of random A sites are expected to fold into 5ʹB-[A–X] motif as the minimum free energy structure. Therefore, while further experiments are needed to assess how m^6^A stabilizes / destabilizes various motifs in different sequence contexts, these results indicate that m^6^A could strongly bias RNA folding toward 5ʹB-[m^6^A–X] motifs, and thus help explain the m^6^A induced RNA conformational changes observed in transcriptome-wide structure mapping data.

### m^6^A is recognized in 5ʹG-[m^6^A–U] but not in duplexes

To examine the functional significance of the 5ʹG-[m^6^A–U] motif, we examined whether it can be recognized by the YTH domain m^6^A reader (residues 380-579) from the human YTHDF2 (NP_057342.2) protein^45^. Binding was measured using a fluorescence polarization (FP) assay employing fluorescein tagged RNA^46^. Experiments were initially carried out in the absence of Mg^2+^ to allow comparison with previous studies^45,47–50^.

**Fig. 4:**
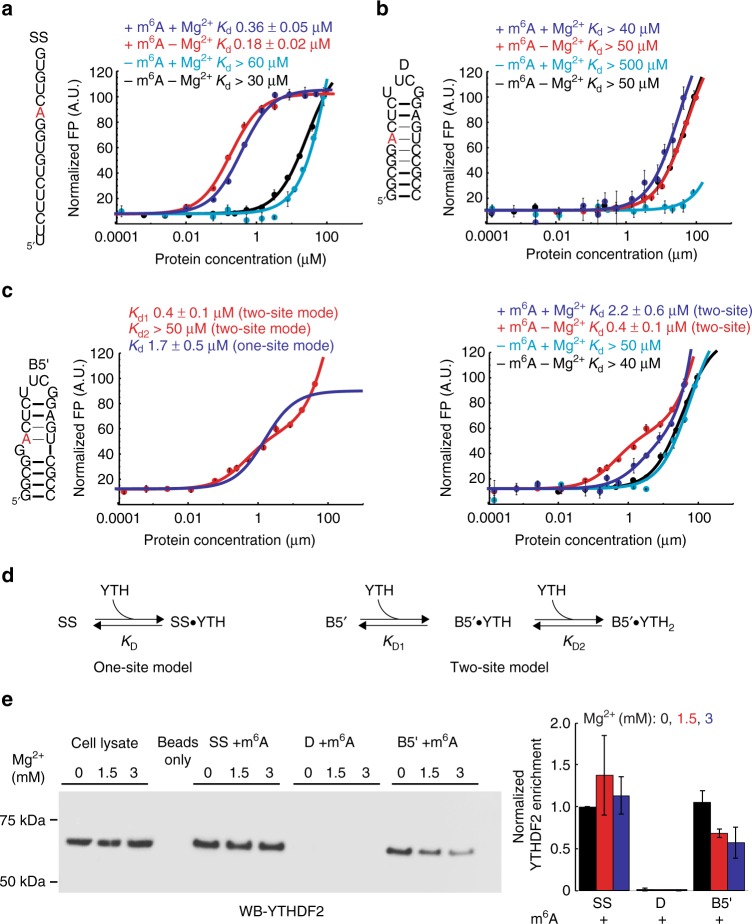
YTH recognizes m^6^A in B5ʹ but not D. **a**–**c** Binding curves for YTH domain with **a** SS, **b** D, and **c** B5ʹ. **d** One-site and independent two-site binding models used for curve fitting. Data is shown for unmodified and methylated RNA with and without 3 mM Mg^2+^. Uncertainty reflects the standard deviation from three measurements. (**c**, left) Fits to the data assuming single (blue) versus two independent (red) binding sites (see Methods). **e** In vitro RNA pulldown assay using 5ʹbiotinylated RNA constructs with m^6^A. Showing YTHDF2 binds preferentially to m^6^A in unpaired context or at the junction, and Mg^2+^ decreases the enrichment of YTHDF2 by methylated B5ʹ. Blots shown are representative of results from three experiments. Uncropped blots are shown in Supplementary Fig. 6. The semi-quantitative YTHDF2 enrichment analysis was performed using ImageJ^56^. The uncertainty represents the standard deviation from three experiments

As a positive control, the YTH domain binds to single-stranded RNA (SS) containing m^6^A and the consensus sequence (GGm^6^ACU) with *K*_D_ = 0.18 ± 0.02 µM (Fig. 4a) in good agreement with previously reported values (0.2-2 µM)^45,47–49^. The binding affinity is diminished at least ~ 100-fold (*K*_D_ > 30 µM) for unmodified SS (Fig. 4a), in good agreement with 10–100-fold weaker affinity reported previously^45,47–49^.

Surprisingly, the YTH domain binds weakly (*K*_D_ > 50 µM) to both unmodified and methylated duplex RNA (D**)** (Fig. 4b). In both cases, the affinity is comparable to that measured for unmodified SS (Fig. 4b). This indicates that m^6^A sites in helical m^6^A–U Watson-Crick bps are not accessible for recognition by the YTH domain. This result is significant considering that helical A–U Watson-Crick bps occur frequently in the structures of RNA.

In contrast, the YTH domain does bind tightly to methylated B5ʹ (but not its unmodified counterpart; *K*_D_ > 40 µM) (Fig. 4c). The binding curve deviates from a simple two-state model and the data is better fit to a model that assumes two independent binding sites (Fig. 4d). A similar behavior was previously observed for YTH domain binding to methylated ssRNA^45^. One of two *K*_D_s obtained from this analysis (*K*_D1_ = 0.3 ± 0.1 µM) is consistent with high affinity binding as observed for methylated SS while the other (*K*_D2_ > 50 µM) is consistent with weak binding as observed with unmodified RNA. It is possible that the YTH domain specifically binds to m^6^A in B5ʹ with high affinity but also weakly and non-specifically binds to other parts of the RNA (e.g. duplex or apical loop region). Alternatively, the YTH domain could bind to m^6^A in two different RNA conformations (e.g. helical versus more single-stranded). These results indicate that in contrast to duplexes, m^6^A in 5ʹB-[m^6^A–U] is recognized by the YTH domain.

Based on the NMR data, Mg^2+^ stabilizes the junctional m^6^A–U bp in B5ʹ potentially making m^6^A6 less accessible for recognition. Indeed, the binding affinity to methylated B5ʹ decreased by ~6-fold (*K*_D1_ 0.4 ± 0.1 µM versus 2.2 ± 0.6 µM, Fig. 4c) in the presence of 3 mM Mg^2+^ but remained ~10 fold tighter than that of unmodified SS or B5ʹ and ~ 20-fold tighter than methylated D. By comparison, the binding affinity of YTH domain to methylated or unmodified SS and D as well as unmodified B5ʹ was only slightly (by ~1-fold) weakened in the presence of Mg^2+^ (Fig. 4a, b).

We confirmed the above results for the full-length YTHDF2 protein using in vitro RNA pulldown experiments (Fig. 4e). Here, biotinylated RNA baits were incubated with HEK293T cell lysates, and RNA:protein complexes were purified with streptavidin agarose. The bound proteins were then eluted and subjected to SDS-PAGE, followed by western blot to detect YTHDF2. In agreement with results from the FP assay, in the absence of Mg^2+^, modified B5ʹ enriches YTHDF2 protein by an amount comparable to modified SS. While similar enrichments were observed in the absence and presence of Mg^2+^ for SS, the enrichment with B5ʹ decreased 1-fold in the presence of Mg^2+^. In sharp contrast, YTHDF2 was barely enriched by methylated D in the absence and presence of Mg^2+^.

**Fig. 5:**
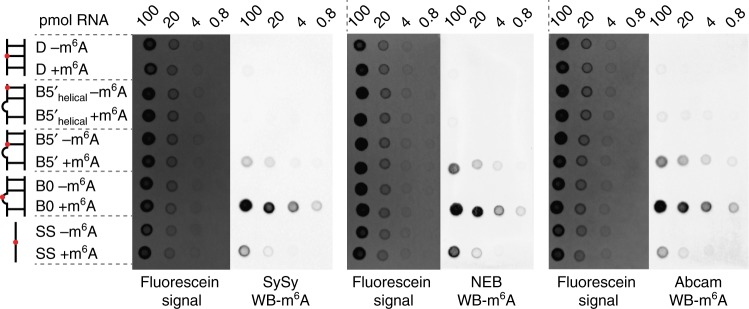
m^6^A antibodies recognize m^6^A in B5ʹ but not D. Dot blot assays assessing the antibody specificity for m^6^A in different secondary structure contexts. Increasing amounts of 5ʹfluorescein labeled RNAs with and without m^6^A were spotted onto the membrane. The quantity of RNA was visualized by the fluorescein signal. Three different m^6^A antibodies (SySy polyclonal, NEB monoclonal, Abcam polyclonal) were tested. Red dots in the schematic secondary structures represent the m^6^A sites. The SS construct used in dot blot assays is 5ʹ-GGCGGm^6^ACUC-3ʹ. Blots shown are representative of results from three experiments

Similar results were also obtained when examining how well m^6^A is recognized by three m^6^A antibodies (Abcam polyclonal antibody, SySy polyclonal antibody and NEB monoclonal antibody) that are used in transcriptome-wide studies. As expected, all unmodified RNA constructs were not well recognized by the antibodies in dot blot assays (Fig. 5). Both methylated SS and B0 exhibited strong signals, suggesting that the antibodies can recognize unpaired m^6^A (Fig. 5 and Supplementary Fig. 5c). Once again, the methylated D and methylated B5ʹ_helical_ were poorly recognized by all three antibodies. This was also the case for a 9-mer duplex lacking the stabilizing UUCG apical loop (Supplementary Fig. 5c). In contrast, the junctional m^6^A–U bp in B5ʹ was well recognized by all three antibodies.

Taken together, these results indicate the m^6^A within the 5ʹG-[m^6^A–U] motif is well recognized by YTH reader proteins as well as commonly used m^6^A antibodies, whereas m^6^A–U bps embedded in duplexes are not.

## Discussion

A growing number of studies indicate that m^6^A can exert biological effects by modulating RNA structure. So far, studies have primarily focused on the destabilization of base pairing due to steric collisions with the methyl group^28,29,31,33^. However, m^6^A has also been shown to stabilize duplexes when placed at dangling ends and within apical loops^28,29^. Our results identify a 5ʹ bulge motif that is predicted to be abundant and stabilized by m^6^A in a Mg^2+^ dependent manner. The 5ʹ bulge most likely provides m^6^A the conformational freedom needed to allow the methyl group to optimally stack with the 3ʹ neighbor while maintaining Watson-Crick m^6^A–U pairing. Mg^2+^ may help to stabilize the resulting backbone conformation at the bulge by neutralizing electrostatic repulsion. In this regard, it is noteworthy that Mg^2+^ preferentially binds to guanine and adenine nucleotides^51^, thus the naturally occurring DRACH motif may enhance the propensity for the 5ʹ neighbor to bulge out. This motif with base-paired m^6^A stacking on its 3ʹ neighbor and with a 5ʹ-neighboring bulged nucleotide can potentially form in a variety of contexts, including bulges, apical loops, and possibly other higher order structures. Further studies are needed to assess the robustness of the observed stabilization across these different structural and sequence contexts.

While biophysical studies show that m^6^A destabilizes pairing of adenine nucleotides in vitro, these results were at odds with in vitro transcriptome-wide nuclease mapping^28^ and SHAPE^35^ data showing that methylation primarily acts to increase the single-stranded character of the purine nucleotide immediately 5ʹ to the m^6^A site, while the adenine nucleotide itself was less affected^28^. These data were obtained devoid of cellular proteins and the conformational differences likely originate directly from the impact of m^6^A on RNA structure. A bias toward the junctional m^6^A bps flanked by a 5ʹ bulge following methylation could help explain the increase in single-stranded character observed specifically for the purine nucleotide 5ʹ to the m^6^A bulge as well as the increase in the double-stranded character observed for the m^6^A nucleotide using transcriptome-wide nuclease mapping experiments^28^. This bias could be significant especially when considering that m^6^A selectively stabilizes the 5ʹB-[A–U] motif but destabilizes other common motifs such as duplexes and bulges. It should be noted, however, that in vivo SHAPE data^35^ suggest that the impact of methylation on RNA structure can be more complex, possibly due to interactions with proteins.

Our results show that m^6^A is effectively recognized by both YTH domain reader proteins and m^6^A antibodies when located in the 5ʹG-[A–U] motif. The motif could therefore also serve functional roles recruiting YTH domains and is likely to be identified in current methods used to map m^6^A transcriptome-wide. In contrast, our results indicate that both YTH domain reader proteins and m^6^A antibodies do not recognize m^6^A–U bps embedded within duplexes. Current methods for m^6^A mapping attempt to remove these structural biases through nuclease digestion and heating of the RNA prior to immunoprecipitation. However, the RNA fragments are still 30–130 nt long^43^ and are subjected to cooling on ice following heating, allowing for re-annealing of the RNA. Indeed, we found that m^6^A sites buried in A–U bps within duplexes were poorly recognized by the antibodies even when increasing the temperature and/or duration of heating with or without 8 M urea. This raises the possibility that there are m^6^A sites buried within duplexes that have evaded detection using current transcriptome-wide methods. This could potentially confound the interpretation of m^6^A induced conformational changes based on transcriptome-wide structure mapping data since motifs in which m^6^A is accessible such as 5ʹG-[A–U] could be over-represented compared with those in which m^6^A is not accessible. Additional studies are needed to examine whether these structure-specific biases in m^6^A recognition also occur during immunoprecipitation experiments with free-floating RNA used in the transcriptome-wide mapping of m^6^A sites.

Our results indicate that while m^6^A destabilizes duplex A–U bps and disrupts base stacking in certain motifs, the structure landscape of the epitranscriptome is also likely punctuated by motifs such as 5ʹG-[m^6^A–U] that are stabilized by m^6^A. This motif presents a readable m^6^A site in a structured region of RNA. While the biological significance of this motif, including whether it is recognized by specific RNA-binding proteins, requires further investigation, our findings expand the mechanisms by which m^6^A can modulate RNA structure and should facilitate the interpretation of transcriptome-wide data.

## Methods

### Sample preparation

Modified (*N*^6^-methylated adenosine, 5ʹ-biotinylated RNA and 5ʹ-fluorescein labeled RNA) and unmodified RNA oligonucleotides were synthesized using a MerMade 6 Oligo Synthesizer employing 2′-tBDSilyl protected phosphoramidite (ChemGenes) on 1 μmol standard synthesis columns (1000 Å) (BioAutomation). n-acetyl protected rC, rA, and rG phosphoramidites were used to avoid incomplete deprotection when using isobutyryl protected rG and benzoyl protected rA phosphoramidites. m^6^A phosphoramidite was purchased from ChemeGenes, biotin phosphoramdites, and fluorescein phosphoramdites were purchased from Glen Research. The ^15^N1-labeled guanosine and the ^15^N3-labeled uridine phosphoramidites were synthesized according to a published procedure^52^. ssRNA oligonucleotides were synthesized with the option to leave the final 5ʹ-protecting group (4,4ʹ-dimethoxytrityl (DMT)) on for 2ʹO deprotection and cartridge purification. UUCG-capped hairpin RNAs were synthesized with DMT group off for DMT-off 2ʹO deprotection and PAGE purification, since regular 2ʹO deprotection method might cause incomplete deprotection for UUCG-capped samples. Synthesized oligonucleotides were cleaved from the 1 μmol column using 1 mL ammonia methylamine (1:1 ratio of 30% ammonium hydroxide and 30% methylamine) followed by 2-hour incubation at room temperature to allow base deprotection. The solution was then air-dried and dissolved in 115 μL DMSO, 60 μL TEA, and 75 uL TEA-3HF for regular 2ʹO deprotection, or in 100 μL DMSO and 125 μL TEA-3HF for DMT-off 2ʹO deprotection, followed by 2.5 h incubation at 65 °C. Regular 2ʹO deprotected samples were then quenched with Glen-Pak RNA quenching buffer and loaded onto Glen-Pak RNA cartridges (Glen Research Corporation) for purification using the online protocol (http://www.glenresearch.com/). Samples were then ethanol precipitated, air dried, dissolved in water and buffer exchanged or diluted into the desired buffer (15 mM sodium phosphate, 25 mM NaCl, 0.1 mM EDTA, 10% D_2_O, pH 6.4 with or without 3 mM Mg^2+^). DMT-off 2ʹO deprotected samples were directly ethanol precipitated, and purified using 20% (w/v) denaturing PAGE, and electroeluted into 20 mM Tris buffer, pH 8, and subsequently ethanol precipitated. The samples were then dissolved in water (50 µM for hairpin and 200–500 µM for duplex), annealed by heating at 95 °C for 10 min, and cooled on ice for 30 min (for hairpin) or at room temperature for 2 h (for duplex). RNA samples were buffer exchanged at least three times using a centrifugal concentrator (EMD Millipore) into the desired buffer. The purity of methylated and unmodified oligonucleotides was verified using NMR to monitor resonances from deprotecting groups and for B5ʹ using liquid chromatography-mass spectrometry LC/MS (Novatia).

### NMR spectroscopy

All NMR experiments were collected on a 600 MHz Bruker NMR spectrometer equipped with an HCN cryogenic probe. Data were processed and analyzed using NMRpipe^53^ and SPARKY (T.D. Goddard and D.G. Kneller, SPARKY 3, University of California, San Francisco), respectively. Resonances were assigned using 2D HSQC, HMQC, ^1^H-^1^H NOESY (mixing times of 100 ms and 150 ms), and HCN experiments in the absence of Mg^2+^.

### UV melting

Thermal melting experiments were conducted on a PerkinElmer Lambda 25 UV/VIS spectrometer with a RTP 6 Peltier Temperature Programmer and a PCB 1500 Water Peltier System. All RNA samples were buffer exchanged at least three times with a centrifugal concentrator (EMD Millipore) to desired buffers (15 mM sodium phosphate, 25 mM NaCl, 0.1 mM EDTA, pH 6.4 with or without 3 mM Mg^2+^), followed by direct dilution to 3 µM with the same buffer. At least three measurements were carried out for each RNA with a sample volume of 400 µL in a Teflon-stoppered 1 cm path length quartz cell. The absorbance at 260 nm was monitored while the temperature was varied between 15 and 95 °C.

Thermodynamic parameters from UV melting experiments were fitted using nonlinear model fitting in Mathematica 10.0 (Wolfram Research) such that melting temperature (*T*_m_) and enthalpy (Δ*H*) for duplex and hairpin association were obtained by the fitting to Eqs. (1) and (2), respectively^54^,

1 $$f_{{\mathrm{duplex}}} = \frac{{1 + 4{\mathrm e}^{(\frac{1}{{T_{\mathrm m}}} \, -\, \frac{1}{T})\frac{{{\mathrm{\Delta }}H}}{R}} - \sqrt {1 + 8{\mathrm e}^{(\frac{1}{{T_{\mathrm m}}} \, -\, \frac{1}{T})\frac{{{\mathrm{\Delta }}H}}{R}}} }}{{4{\mathrm e}^{(\frac{1}{{T_{\mathrm m}}} \, -\, \frac{1}{T})\frac{{{\mathrm{\Delta }}H}}{R}}}}$$

2 $$f_{{\mathrm{hairpin}}} = \frac{{{\mathrm e}^{(\frac{1}{{T_{\mathrm m}}} \, -\, \frac{1}{T})\frac{{{\mathrm{\Delta }}H}}{R}}}}{{1 + {\mathrm e}^{(\frac{1}{{T_{\mathrm m}}} \, -\, \frac{1}{T})\frac{{{\mathrm{\Delta }}H}}{R}}}}$$

where *T* is the temperature (K), *R* is the gas constant (kcal mol^−1^), and *f* is the fraction of folded duplex or hairpin for Eqs. (1) and (2), respectively. Δ*G* and Δ*S* were calculated from Eqs. (3) and (4) for duplex association and hairpin folding respectively.

3 $$\Delta S = \frac{{\Delta H}}{{T_{\mathrm m}}} - R{\rm ln}(\frac{{C_{\mathrm T}}}{2});\,\Delta G = \Delta H - T\Delta S$$

4 $$\Delta S = \frac{{\Delta H}}{{T_{\mathrm m}}};\,\Delta G = \Delta H - T\Delta S$$

where *C*_T_ is the total concentration of RNA. The uncertainty in *T*_m_, Δ*H*, Δ*G*, and Δ*S* was obtained based on the standard deviation in triplicate measurements. The destabilization or stabilization effects of m^6^A was calculated using the following equations:

5 $$\Delta \Delta G = \Delta G_{\rm m6A} - \Delta G_{\mathrm{unmodified}}$$

6 $$\Delta \Delta H = \Delta H_{\rm m6A} - \Delta H_{\mathrm{unmodified}}$$

7 $$\Delta \Delta S = \Delta S_{\rm m6A} - \Delta S_{\mathrm{unmodified}}$$

### Secondary structure prediction

The programs MC-Flashfold package^39^ and RNAstructure package^44^ were used to predict the secondary structure of 140,574 sequences containing m^6^A that were identified using single-nucleotide transcriptome-wide m^6^A mapping of human genome build (hg19)^43^. Another 140,574 control sequences that do not contain the DRACH motif and that were randomly selected from the same transcriptome were also analyzed. In-house Python scripts were used to perform the analysis. The sequences were 41 nt long with m^6^A positioned in the middle. Analysis was repeated when varying the position and length of the m^6^A site (31-nt long sequences with m^6^A site at the eleventh or twentyfirst position). The lowest energy predicted structures were classified into different categories: duplex, unpaired, 5ʹB-[A–U], 5ʹB-[A–Y] (Y denotes A or C or G), [A–U]-B3ʹ, [A–Y]-B3ʹ and 5ʹB-[X–A]/[X–A]-B3ʹ (X denotes A or C or G or U). 5ʹ-B-[A–U] and 5ʹB-[A–Y] are motifs containing junctional A–U or A–Y bp with unpaired residues at 5ʹ side of A. Similarly, [A–U]-B3ʹ, [A–Y]-B3ʹ are motifs containing junctional A–U or A–Y bps with unpaired residues at 3ʹ side of A. 5ʹB-[X–A]/[X–A]-B3ʹ is the motif with junctional A–X bps that are on the opposite strand of unpaired residues. With the exception of G–U bps, the RNAstructure package does not predict mismatches. To calculate the population of 5ʹB-[A–Y] and [A–Y]-B3ʹ, any A–Y site that was immediately adjacent to a canonical Watson-Crick bp was counted as a mismatch. The MC-Flashfold package does predict mismatches allowing further classification of the duplex category into duplex (A–U) and duplex (A–Y). MC-Flashfold was used to predict the secondary structures setting the maximum energy difference of output structures to minimum free energy (MFE) of 0, 1, 2 or 3 kcal mol^−1^. For RNAstrutcure package, the maximum percent energy difference between predicted structures and MFE was set to 10, 20, or 30%. The population of 5ʹB-[A–U] or 5ʹB-[A–X] was determined to be the number of sequences predicted to form 5ʹB-[A–U] or 5ʹB-[A–X] in the output structures divided by the total number of sequences (140,574).

### Protein expression and purification

A gene, codon optimized (Supplementary Table 2) for *Escherichia coli* expression, encoding the YTH domain (residues 380–579) of human YTHDF2 (NP_057342.2) was subcloned into the pET15b vector (the plasmid containing the codon optimized sequence was purchased from GenScript). The plasmid was transformed into *E. coli* strain C41(DE3). The cells were grown in LB medium containing 50 µg ml^−1^ ampicillin at 37 °C to an OD_600_ between 0.4 and 0.6. Recombinant proteins were induced by adding isopropyl-β-d-thiogalactopyranoside (IPTG) to 0.5 mM and overexpressed in LB medium at 20 °C overnight. Cells expressing YTH domain were harvested at 4 °C and stored at −80 °C or lysed immediately using a microfluidizer in the buffer containing 20 mM HEPES (pH 7.4), 200 mM NaCl, 1 mM DTT supplemented with protease inhibitors and DNase. Following centrifugation at 17,500 rpm for 3 min at 4 °C, the supernatant was loaded to Ni-NTA affinity column. The eluted protein was further purified by size exchange chromatography (SEC) using a Superdex 75 pg column. The peak fractions were collected and concentrated. The protein purity was assessed using SDS-PAGE analysis and protein concentration determined using Bradford assays.

### YTH domain binding assays

Binding experiments were carried out using a previously reported^46^ fluorescence polarization (FP) assay at 25 °C and using a PanVera Beacon 2000 instrument (Invitrogen, Madison, WI, USA). 5ʹ-fluorescein-labeled RNAs were synthesized and purified as described in the sample preparation section. Fluorescein labeled samples were dissolved in water and then directly diluted into the desired buffer (20 mM HEPES 50 mM NaCl, 3 mM DTT, pH 8.2, with or without 3 mM Mg^2+^). YTH domain protein was serially diluted into 200 µL of the same binding buffer containing 2 nM 5ʹ-fluorescein-labeled RNA. Fluorescence polarization was measured at an excitation wavelength of 490 nm and an emission wavelength at 530 nm. The total fluorescent intensities did not vary significantly throughout the measurements (i.e., (*I*_max_ − *I*_min_)/*I*_min_ ~5%, in which *I*_max_ and *I*_min_ are the maximum and minimum intensities, respectively). The binding curves were fitted to either one-site (Eq. (8)) or two-site^55^ (Eq. (9)) binding mode using Mathematica 10.0 (Wolfram Research).

8 $$A_{\mathrm t} = \frac{{A - (A - B) \times (R_{\mathrm{t}} + L_{\mathrm{t}} + K_{\mathrm{D}} - \sqrt {\left( {R_{\mathrm{t}} + L_{\mathrm{t}} + K_{\mathrm{D}}} \right)^2 - 4 \times R_{\mathrm{t}} \times L_{\mathrm{t}}} )}}{{2 \times R_t}}$$

9 $$A_{\mathrm t} = \frac{{A + \frac{{B \times L}}{{K_{{\mathrm{D}}1}}} + \frac{{C \times L^2}}{{K_{{\mathrm{D}}1} \times K_{{\mathrm{D}}2}}}}}{{1 + \frac{L}{{K_{{\mathrm{D}}1}}} + \frac{{L^2}}{{K_{{\mathrm{D}}1} \times K_{{\mathrm{D}}2}}}}}$$

*L*_t_ is the total concentration of protein, *R*_t_ is the concentration of RNA. *A* and *B* represent free RNA anisotropy and RNA-protein intrinsic anisotropy of the saturated protein-RNA complex. *K*_D_ is the dissociation constant. In Eq. (9), *K*_D1_ and *K*_D2_ are dissociation constants of the two binding events respectively. *A*, *B*, and *C* represent free RNA anisotropy, RNA-Protein and RNA-[Protein]_2_ intrinsic anisotropy of the saturated protein-RNA complexes. By titrating excess protein against 2 nM RNA, *L*_free_ remains in excess over concentrations of RNA-Protein and RNA-[Protein]_2_ complexes. *L*_free_ is therefore well approximated by *L*_t_ and is referred to simply as *L*.

### **In vitro** RNA pulldown

Biotinylated RNA samples were synthesized and purified as described in the sample preparation section. HEK293T cells were harvested at ~70–80% confluency, washed with cold PBS and lysed by dounce homogenization in lysis buffer (10 mM NaCl, 2 mM EDTA, 0.5% Triton X-100, 0.5 mM DTT, 10 mM Tris, pH 7.4), and the lysate was then brought to 150 mM KCl and 5% Glycerol (v/v). Mammalian protease inhibitor cocktail (Sigma-Aldrich) and phosphatase inhibitor cocktail (Sigma-Aldrich) were freshly added to the lysis buffer. The following steps were performed as described previously^5^, except that binding buffers containing different Mg^2+^ concentrations were used (10 mM Tris pH 7.5, 150 mM KCl, 0.5 mM DTT, 0.05% (v/v) NP-40 and 0 mM, 1.5 mM, or 3 mM MgCl_2_). Proteins were eluted under mild conditions with elution buffer (50 mM Tris, pH 7.5, 200 mM NaCl, 2% SDS (w/v), and 1 mM biotin) at 60 °C, 1100 rpm on a thermal block shaker. The pulldown eluent was loaded on 4–12% polyacrylamide Bis-Tris polyacrylamide gels (Thermofisher) and then transferred onto PVDF membranes (GE Healthcare, Amersham) using a wet electrophoretic transfer system (BioRad). The membrane was then blocked with 5% nonfat dry milk in 0.1% PBST (0.1% Tween-20 in 1× PBS, pH 7.4). Rabbit polyclonal YTHDF2 antibody (Aviva system biology ARP67917_P050) was diluted 1:1000 in 0.1% PBST and incubated on the membrane for 1 h at room temperature (15–25 °C) or overnight at 4 °C. Membranes were then washed in 0.1% PBST, and then incubated with HRP-conjugated goat anti-rabbit IgG (Abcam ab6721) at 1:2500 dilution in 0.1% PBST for 1 h at 25 °C. Membranes were then subsequently washed with 0.1% PBST and exposed with enhanced chemiluminescence (ECL; GE Healthcare).

### Dot blot assays

RNA samples were quantified using UV spectroscopy. Methylated and unmodified RNA samples were spotted onto a nylon membrane (GE healthcare). Fluorescein labeled RNA samples (prepared as described in sample preparation section) were scanned by UVP imaging system to visualize the quantity of RNA spotted onto the membrane. The membrane was then UV-crosslinked and blocked for 1 h in 5% nonfat dry milk in 0.1% PBST (0.1% Tween-20 in 1× PBS, pH 7.4). The following steps were performed essentially as described for RNA pulldown (above). m^6^A antibody (SySy 202003, NEB E1610S, or Abcam ab151230) was diluted 1:1000 in 0.1% PBST and incubated on the membrane for 1 h at room temperature or overnight at 4 °C. Membranes were then washed in 0.1% PBST, incubated with HRP-conjugated goat anti-rabbit IgG (Abcam ab6721) (1:2500) in 0.1% PBST for 1 h at room temperature. The membrane was then washed again in 0.1% PBST, and developed with enhanced chemiluminescence (ECL; GE Healthcare). The **SS** construct used in dot blot assay is 5ʹ-GGCGGm^6^ACUC-3ʹ.

### Code availability

The in-house Python scripts used for secondary structure prediction are available on request to the corresponding author.

### Data availability

The authors declare that the data supporting the findings of this study are available within the article and its Supplementary Information files, or are available upon reasonable requests to the authors.

## Electronic supplementary material

Supplementary Information

Peer Review File
