# Supplementary material for: A potentially abundant junctional RNA motif stabilized by m6A and Mg2+
Source: Nat Commun. 2018 Jul 17;9:2761. doi: 10.1038/s41467-018-05243-z (PMC6050335; doi:10.1038/s41467-018-05243-z)
Supplement: Supplementary file 1 — Supplementary Information [file 41467_2018_5243_MOESM1_ESM.pdf]

Supplementary Information

A potentially abundant junctional RNA motif stabilized by m^6A and Mg^{2+}

Liu et al.

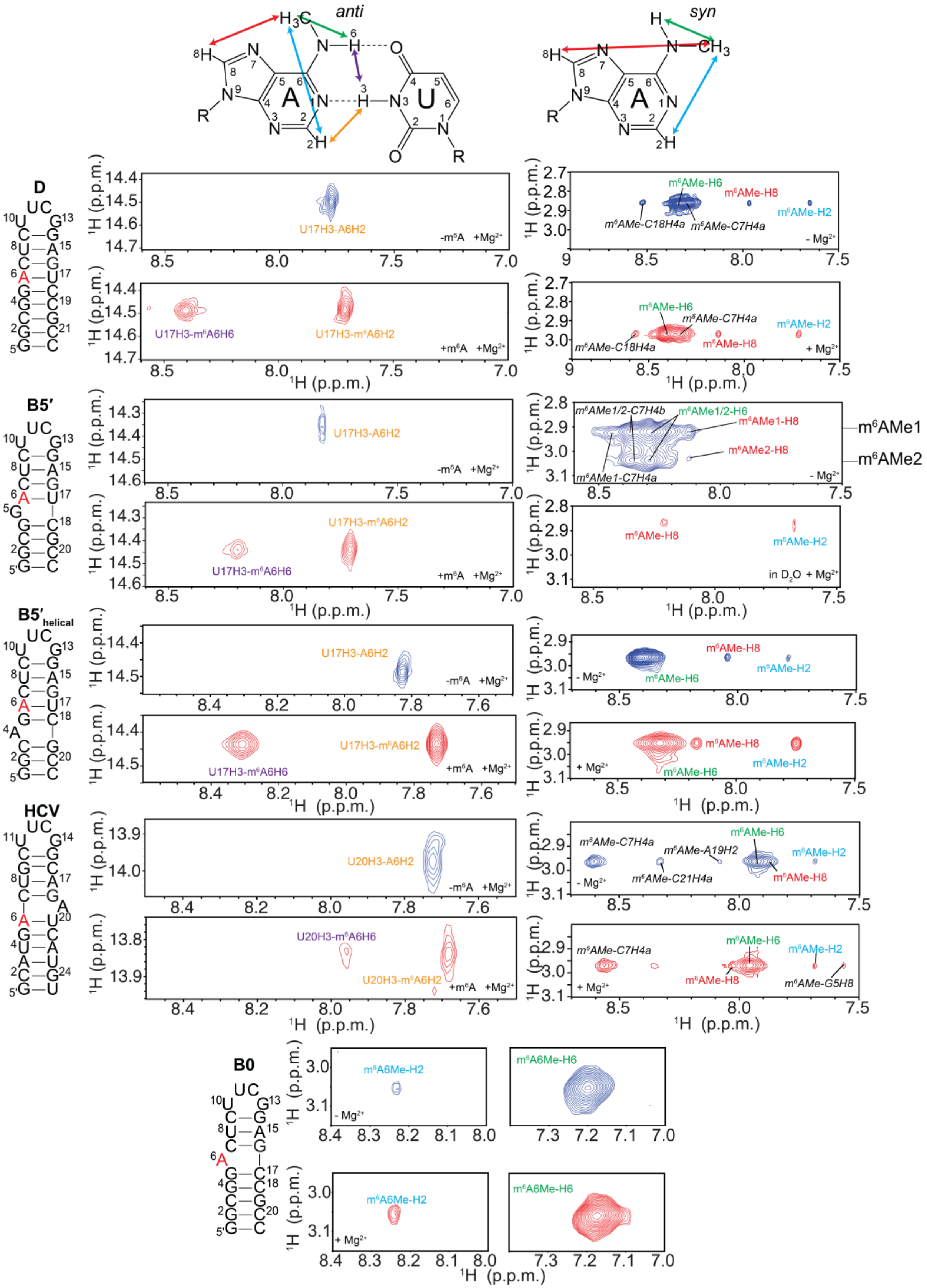

anti
syn
D
B5′
B5′helical
HCV
B0
U17H3-A6H2
U17H3-m6A6H6
U17H3-m6A6H2
U20H3-A6H2
U20H3-m6A6H6
U20H3-m6A6H2
m6AMe-H6
m6AMe-H8
m6AMe-H2
m6AMe-C18H4a
m6AMe-C7H4a
m6AMe1
m6AMe2
m6AMe1-H8
m6AMe2-H8
m6AMe1/2-H6
m6AMe1/2-C7H4b
m6AMe1-C7H4a
m6AMe-A19H2
m6AMe-C21H4a
m6AMe-G5H8
m6A6Me-H2
m6A6Me-H6
in D2O + Mg2+
1H (p.p.m.)

Supplementary Figure 1. Analysis of the m^6A methyl conformation in D, B5', $B5'_{helical}$, HCV and B0. 2D Nuclear Overhauser Effect Spectroscopy (NOESY) spectra showing distance-based connectivity involving the N^6-methyl proton in D and B5' in the presence and absence of Mg^{2+}. Shown are color coded distances and NOE cross peaks between protons that are sensitive to the conformation (*syn* versus *anti*) of the N^6-methyl proton in m^6A and m^6A-U base pairing.

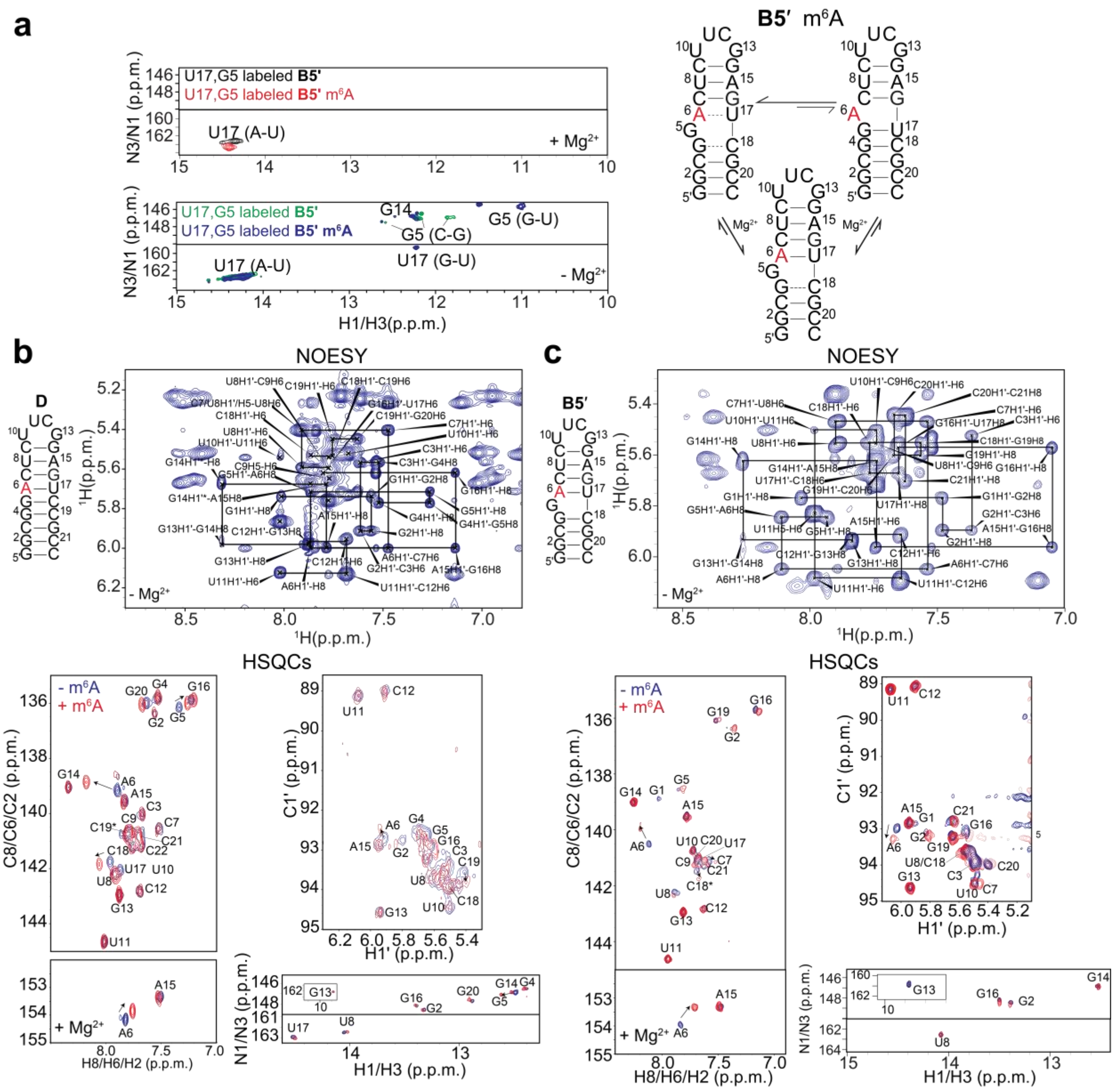

Supplementary Figure 2. Resonance assignment and NMR spectra for D and B5'. (a) 2D NH Heteronuclear single quantum coherence spectroscopy (HSQC) spectra of ^{15}N site-labeled (U17N3 + G5N1) B5' with and without the m^{6}A6 modification in the presence and absence of 3 mM Mg^{2+}. Non-exchangeable proton region of 2D NOESY spectra for unmodified D (b) and B5' (c) in the absence of Mg^{2+}, and assigned 2D HSQC spectra for D (b) and B5' (c) with and without m^{6}A in the presence of Mg^{2+}.

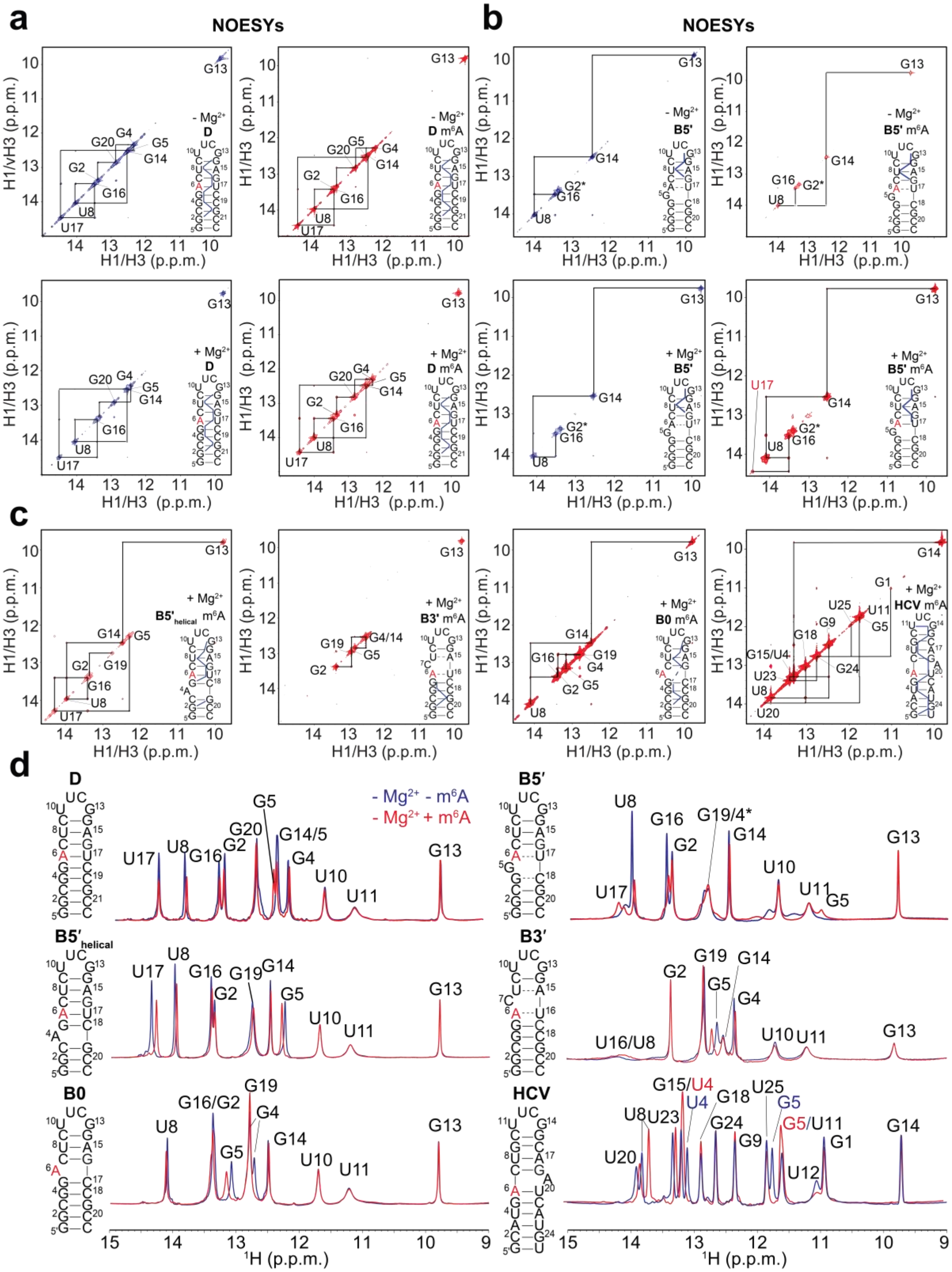
a
NOESYs
b
NOESYs
c
d
H1/vH3 (p.p.m.)
H1/H3 (p.p.m.)
- Mg2+
+ Mg2+
D
D m6A
B5'
B5' m6A
B5'helical m6A
B3' m6A
B0 m6A
HCV m6A
- Mg2+ - m6A
- Mg2+ + m6A
D
B5′
B5′helical
B3′
B0
HCV
1H (p.p.m.)

Supplementary Figure 3. Imino resonance assignment for D, B5', B5'$_{helical}$, B3', B0, and HCV. 2D NOESY spectra showing sequential connectivity between imino protons for (a) D, (b) B5' with and without m^6A in the absence and presence of 3mM Mg^{2+}, and for (c) methylated B5'$_{helical}$, B3', B0, HCV in the presence of Mg^{2+}. (d) ^1H spectra of D, B5', B5'$_{helical}$, B3', B0, HCV with and without m^6A in the absence of Mg^{2+}.

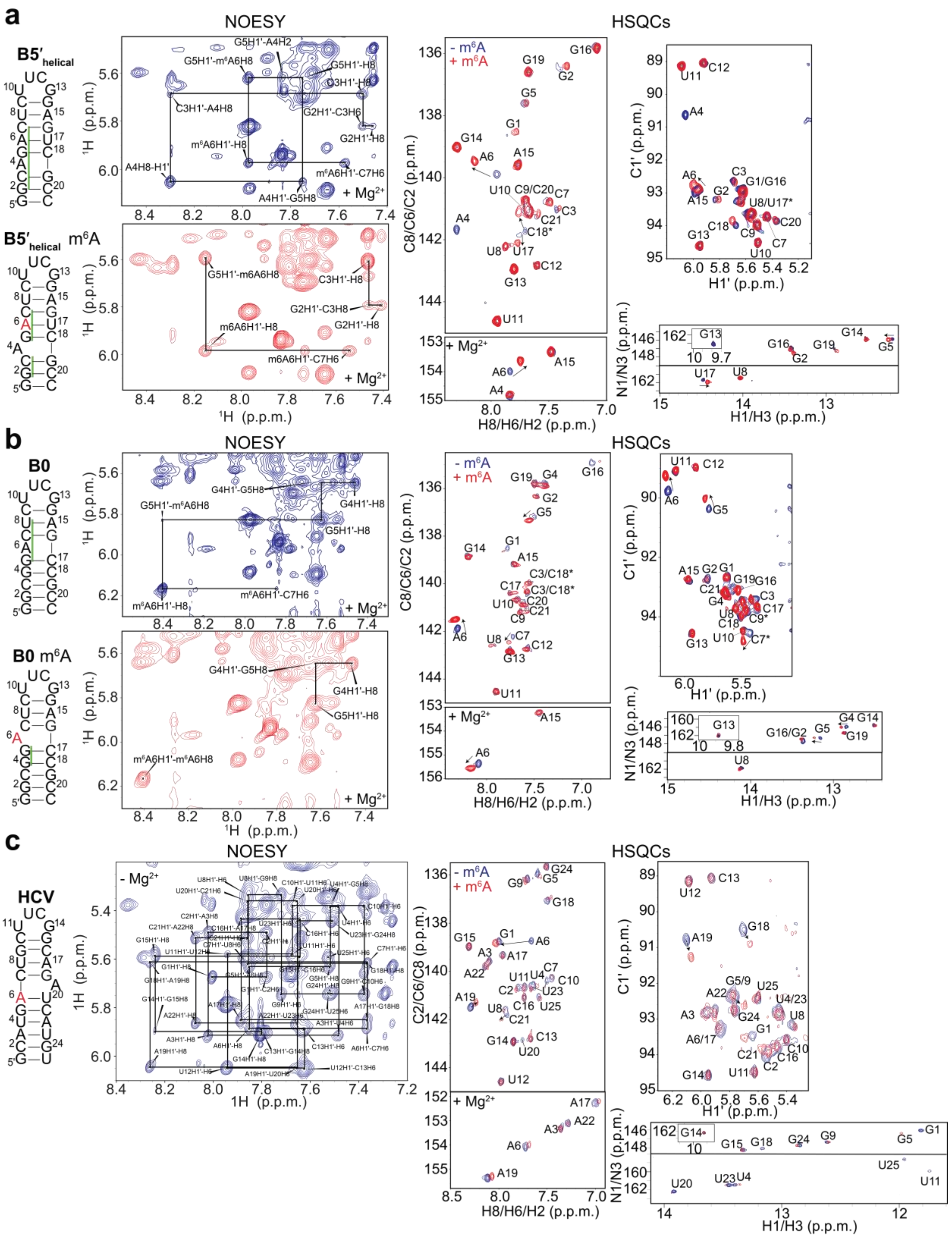
a
NOESY
HSQCs
B5′helical
B5′helical m6A
+ Mg2+
- m6A
+ m6A
1H (p.p.m.)
C8/C6/C2 (p.p.m.)
H8/H6/H2 (p.p.m.)
C1′ (p.p.m.)
H1′ (p.p.m.)
N1/N3 (p.p.m.)
H1/H3 (p.p.m.)
b
NOESY
HSQCs
B0
B0 m6A
c
NOESY
HSQCs
HCV
- Mg2+
C2/C6/C8 (p.p.m.)

Supplementary Figure 4. Resonance assignment and NMR spectra for $B5'_{helical}$, B0 and HCV. Non-exchangeable proton region of 2D NOESY spectra, and 2D HSQC spectra for $B5'_{helical}$ (a) and B0 (b) with and without m^6A in the presence of Mg^{2+}. (c) Non-exchangeable proton region of 2D NOESY spectra for HCV in the absence of Mg^{2+}, and 2D HSQC spectra for HCV with and without m^6A in the presence of Mg^{2+}.

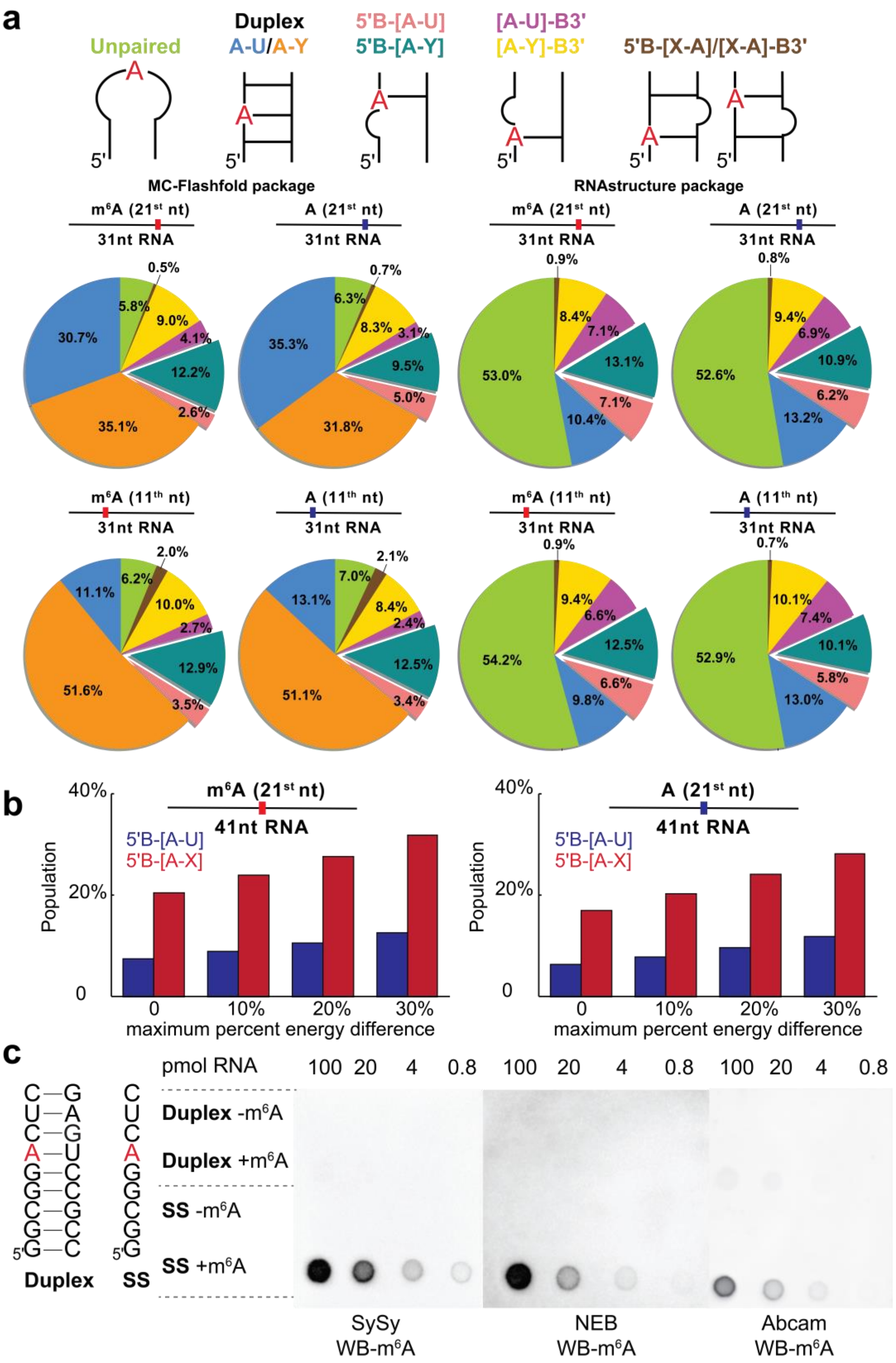

a
Unpaired
Duplex
A-U/A-Y
5'B-[A-U]
5'B-[A-Y]
[A-U]-B3'
[A-Y]-B3'
5'B-[X-A]/[X-A]-B3'
MC-Flashfold package
RNAstructure package
m6A (21st nt)
A (21st nt)
31nt RNA
m6A (11th nt)
A (11th nt)
b
m6A (21st nt)
A (21st nt)
41nt RNA
5'B-[A-U]
5'B-[A-X]
Population
maximum percent energy difference
c
pmol RNA
100 20 4 0.8
Duplex -m6A
Duplex +m6A
SS -m6A
SS +m6A
Duplex
SS
SySy
WB-m6A
NEB
WB-m6A
Abcam
WB-m6A

Supplementary Figure 5. Secondary structure prediction of m^{6}A sites in the transcriptome and m^{6}A antibody specificity assay. (a) Secondary structure context of 140,574 m^{6}A sites mapped using individual-nucleotide-resolution cross-linking and immunoprecipitation (miCLIP)[1] in the human transcriptome predicted using RNAstructure[2] and MC-Flashfold[3]. Also shown are corresponding predictions for 140,574 unmodified adenine sites selected randomly from the same human transcriptome. Secondary structures were predicted for RNA sequences of 31-nt long with m^{6}A (or A) at the 11th position (a) or 21st position. Shown is the distribution for the lowest energy predicted structures, classified according to the m^{6}A secondary structural context: Junction Watson-Crick m^{6}A-U or mismatch m^{6}A-Y (Y denotes A or C or G.) with the bulge located 5' (5'B-[A-U] and 5'B-[A-Y]) or 3' ([A-U]-B3' and [A-Y]-B3') to m^{6}A; or with the bulge located 5' or 3' (5'B-[X-A]/[X-A]-B3', X denotes A or C or U or G) to m^{6}A partner nucleotide. (b) Population of m^{6}A and A sites predicted (RNAstructure) to fold into the 5'B-[A-U] and 5'B-[A-X] conformation within different energy threshold relative to the lowest energy structure. (c) Dot blot assays using SySy, NEB and Abcam m^{6}A antibodies. Unmodified and methylated 9-mer duplexes lacking the UUCG apical loop and its corresponding ssRNA were tested. Blots shown are representative of results from three experiments.

Figure 4e

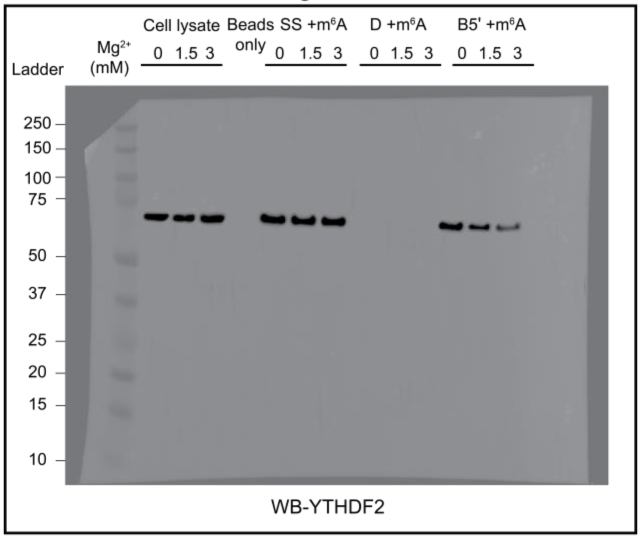

Figure 5

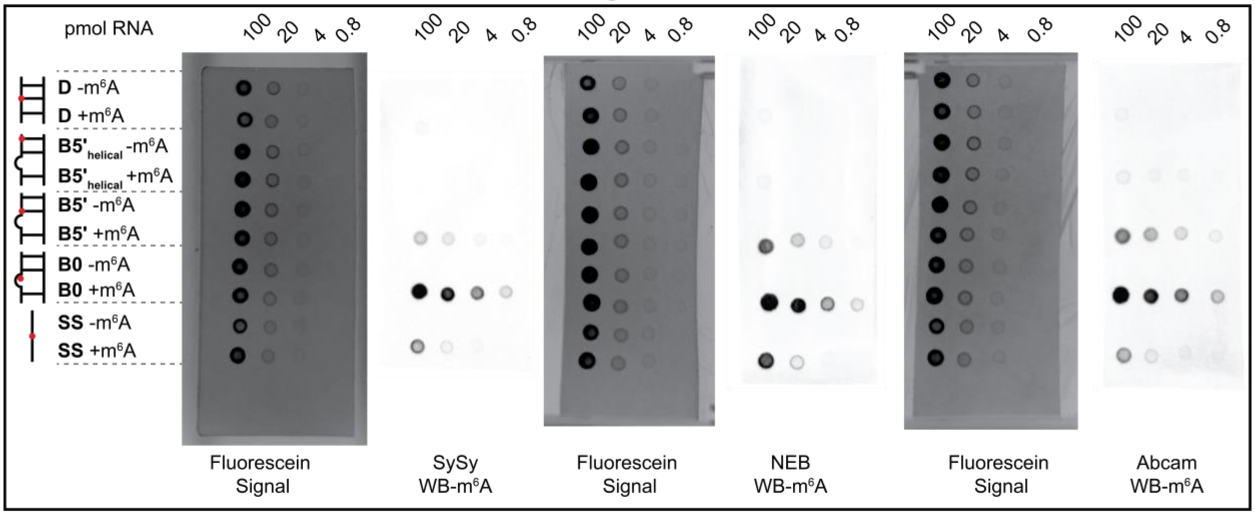

Figure S5c

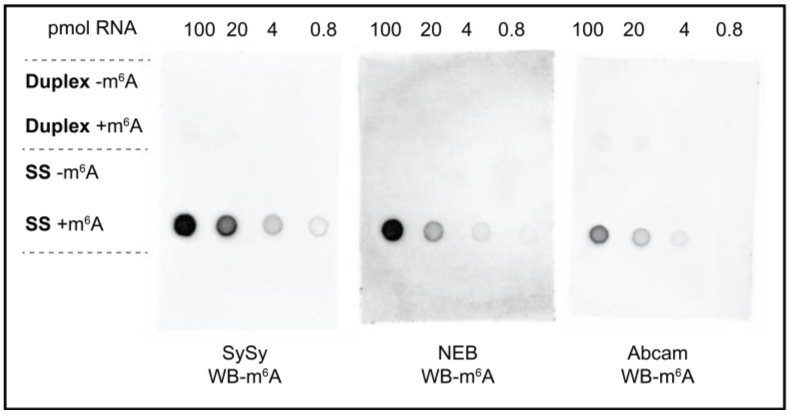

Supplementary Figure 6. Uncropped blots.

Supplementary Table 1. RNA thermodynamic parameters from UV melting

Construct	n	[Mg2+] (mM)	Ct (μM)	Tm(°C)	ΔH (kcal mol^{-1})	ΔS (e.u.)	$ΔG_{37°C}$ (kcal mol^{-1})
D*	3	0	3	54.6±0.2	-86.1±1.5	-236.1±4.7	-12.9±0.1
D* m^6A	3	0	3	51.2±0.2	-87.7±1.3	-243.7±4.0	-12.10±0.02
D*	3	3	3	66.0±0.4	-94.9±1.1	-253.2±3.0	-16.4±0.2
D* m^6A	3	3	3	63.4±0.1	-95.5±2.2	-257.0±6.6	-15.8±0.2
B5'	3	0	3	79.3±0.1	-63.2±1.4	-179.4±3.9	-7.6±0.2
B5' m^6A	3	0	3	76.2±0.1	-70.7±1.2	-202.5±3.7	-7.9±0.1
B5'	3	3	3	82.8±0.5	-64.8±0.5	-182.1±1.3	-8.3±0.1
B5' m^6A	3	3	3	79.8±0.1	-76.7±0.8	-217.2±2.3	-9.2±0.1
$B5'_{helical}$	4	0	3	77.3±0.5	-60.7±2.9	-173.3±8.5	-7.0±0.3
$B5'_{helical}$ m^6A	4	0	3	73.8±0.2	-69.4±0.9	-200.1±2.7	-7.4±0.1
$B5'_{helical}$	4	3	3	81.2±0.3	-63.6±1.2	-179.6±3.7	-7.9±0.1
$B5'_{helical}$ m^6A	4	3	3	77.8±0.4	-71.1±2.8	-202.6±7.9	-8.3±0.3
B0	4	0	3	79.2±0.4	-57.5±1.6	-163.3±4.5	-6.9±0.2
B0 m^6A	4	0	3	78.0±0.2	-56.1±1.4	-159.8±3.9	-6.5±0.2
B0	4	3	3	89.0±1.0	-50.8±1.0	-140.3±3.2	-7.29±0.03
B0 m^6A	4	3	3	86.5±0.6	-48.2±2.1	-134.2±5.6	-6.6±0.3
B3'	3	0	3	75.1±0.3	-72.2±1.8	-207.4±5.3	-7.9±0.1
B3' m^6A	3	0	3	71.3±0.5	-65.6±1.0	-190.4±2.6	-6.5±0.2
B3'	3	3	3	87.9±0.2	-44.6±0.6	-123.6±1.6	-6.3±0.1
B3' m^6A	3	3	3	86.2±0.2	-37.2±1.0	-103.5±2.8	-5.1±0.1
HCV	3	0	3	66.9±0.9	-48.4±1.6	-142.4±4.4	-4.3±0.2
HCV m^6A	3	0	3	62.5±0.6	-37.9±1.6	-112.9±4.5	-2.9±0.2
HCV	3	3	3	75.4±0.2	-43.6±2.6	-125.2±7.6	-4.8±0.3
HCV m^6A	3	3	3	71.1±0.6	-44.5±1.2	-129.4±3.5	-4.4±0.1

n = number of independent measurements. Data are presented as the mean ± standard deviation (s.d.). Uncertainty in the calculated thermodynamic parameters were determined by error propagation as described previously (Siegfried, N.A. *et al.*, *Biochemistry.* **46**, 172–181, 2007). * D constructs used here are the duplex version

(without UUCG apical loop) since T_m (melting temperature) of UUCG-D is above 90 °C which cannot be accurately measured.

Supplementary Table 2. Condon-optimized sequence for YTH domain of YTHDF2

CATATGAGCACCCCGAGCGAGCCGCATCCGGTTCTGGAGAAACTGCGTAGCAT<br>CAATAACTATAACCCGAAGGACTTTGACTGGAATCTGAAGCACGGTCGTGTGTTC<br>ATCATTAAAAGCTACAGCGAGGACGATATCCACCGTAGCATTAAGTATAACATCT<br>GGTGCAGCACCGAACACGGCAACAAACGTCTGGACGCGGCGTACCGTAGCATG<br>AACGGCAAGGGCCCGGTGTATCTGCTGTTCAGCGTTAACGGTAGCGGCCACTTT<br>TGCGGTGTGGCGGAGATGAAAAGCGCGGTTGATTACAACACCTGCGCGGGTGT<br>GTGGAGCCAGGACAAGTGGAAAGGCCGTTTCGATGTTCGTTGGATTTTTGTGAA<br>AGACGTTCCGAACAGCCAACTGCGTCACATCCGTCTGGAGAACAACGAAAACAA<br>GCCGGTTACCAACAGCCGTGATACCCAGGAAGTGCCGCTGGAAAAGGCGAAAC<br>AAGTTCTGAAGATCATTGCGAGCTACAAACACACCACCAGCATTTTCGACGATTT<br>TAGCCACTATGAAAAACGTCAAGAGGAAGAAGAAAGCGTGAAAAAGGAGCGTCA<br>AGGTCGTGGTAAATAACTCGAG

Supplementary Note. NMR analysis of RNA constructs

If the N^6-methyl group in m^6A adopts a *syn* conformation, distance-based Nuclear Overhauser effect (NOE) cross-peaks are expected between the methyl proton and m^6A-H2 (distance ~4.8Å) whereas no NOE cross-peaks are expected between the methyl proton and m^6A-H8 (distance ~7Å) (Supplementary Fig. 1). In contrast, for an *anti* conformation, a stronger m^6A methyl proton-H8 NOE cross peak is expected (distance ~4.7Å) as compared to m^6A methyl proton-H2 (distance ~6.0Å) (Supplementary Fig. 1). In the case of D, $B5'_{helical}$ and HCV in the absence and presence of Mg^{2+}, we observe both m^6A methyl proton-H2 and m^6A methyl proton-H8 NOE cross peaks with the latter being slightly stronger (1.1x –1.4x) as expected for a Watson-Crick H-bonded m^6A-U bp. It is possible that the methyl conformation is slightly shifted toward the *syn* orientation.

Alternatively, the amino group might experience fast exchange between the *anti* and *syn* conformations. Two methyl resonances are observed for B5' in the absence of Mg^{2+}, both of which only show the m^{6}A methyl proton-H8 NOEs expected for an *anti* conformation (note that the m^{6}A methyl proton-H2 may not be observable due to exchange broadening (Supplementary Fig. 1)). The intense m^{6}A methyl proton-H2 NOE and upfield shifted amino resonance (~7.2 ppm) in B0 indicate a *syn* conformation for the N^6-methyl group as expected for a bulged nucleotide not involved in base pairing in the absence and presence of Mg^{2+}(Supplementary Fig. 1). The NOE connectivity indicates that in unmodified B0, A6 is flipped in and stacks on G5 and C7. The NOE connectivity is lost in methylated B0. (Supplementary Fig. 4b). This combined with significantly upfield shifted m^{6}A6-C1' (Supplementary Fig. 4b) indicates that m^{6}A adopts a flipped out conformation. It is possible that the methyl group forms sterically unfavorable contacts with its neighbors when m^{6}A is intra-helical, resulting in the loss of favorable stacking. In B5'$_{helical}$, the NOE connectivity between the bulged A4 residue and its neighboring bps is also lost in the methylated RNA, indicating that m^{6}A promotes the flipping out of the A4 bulge (Supplementary Fig. 4a).

Supplementary References

1. Linder, B. et al. Single-nucleotide-resolution mapping of m^{6}A and m^{6}A$_m$ throughout the transcriptome. *Nat. Methods* **12**, 767-72 (2015).
2. Reuter, J.S. & Mathews, D.H. RNAstructure: software for RNA secondary structure prediction and analysis. *BMC Bioinformatics* **11**, 129 (2010).

3. Parisien, M. & Major, F. The MC-Fold and MC-Sym pipeline infers RNA structure from sequence data. *Nature* **452**, 51-5 (2008).
